# State of nutrition amongst US college students: dataset of a national survey study

**DOI:** 10.1186/s13104-023-06273-7

**Published:** 2023-02-02

**Authors:** Saipriya Gande, Rohan K. Mangal, Thor S. Stead, Latha Ganti

**Affiliations:** 1 Academy at the Lakes, Lutz, FL USA; 2 University of Miami Miller School of Medicine, Miami, FL USA; 3 The Warren Alpert School of Medicine, Providence, RI USA; 4 University of Central Florida College of Medicine, Orlando, FL USA

**Keywords:** Nutrition, Daily calories, College students, Mindset towards nutrition, Healthy food expenses

## Abstract

**Objective:**

This article presents the dataset titled “Nutrition habits amongst college students in the United States. [1]” The dataset contains the survey responses of 200 US college students aged 18–24 years regarding their knowledge, attitudes, and challenges with regard to nutrition. The recommended USDA (US daily allowance) is 200 calories, comprised of 2 cups fruits, 2.5 cups vegetables, 5.5 ounces of protein, 6 ounces of grains, and 3 cups of dairy [2]. Adhering to these nutritional guidelines however can be complicated by rising tuition prices, food insecurity, and inability to make one’s own food.

**Data description:**

The data in the dataset attempt to estimate the incidence of these barriers. These data could be useful to understand the barriers to healthy eating amongst young adults, and design targeted solutions.

## Objective

The objective of this research is to survey US College students on their knowledge and habits towards nutrition. It is known that “eating healthy” can actually be difficult, and college students often have special challenges such as being responsible for their own nutrition perhaps for the first time as young adults. The study aimed to decipher whether factors such as knowledge gaps regarding health benefits of a balanced diet exist. The study also aimed to assess whether rising college tuition imposed a barrier and the level of food insecurity among college students.

## Data description

Two hundred (N = 200) domestic U.S. college students ages 18–24 attending a 4-year university were surveyed through a third party anonymous survey research platform. The survey research platform uses organic sampling built on Random Device Engagement (RDE). Using artificial intelligence (AI) to track unique respondent identification, RDE reaches users in their natural environments as they participate in their daily activities through any device. The advanced AI technology and algorithm prevents fraud from single users on multiple accounts and suspicious or illogical responses to specific questions.

One screening question was used to determine survey eligibility. This question asked whether and what type of college student the respondent was. I order to be eligible, the respondent had to be between 18 and 24 years old, and attending a 4-year university in the United States (US).

The survey then consisted of 10 questions. For some of the questions, multiple selections amongst the multiple choices were allowed, so that percentage totals could exceed 100%. The final question was an open-ended one designed to capture the students’ verbatim feelings.

There are two files included in the repository (Table 1). The first is the actual survey instrument used to collect the data. The second file is the actual dataset. The dataset contains the following demographic information: age range, sex, race, US state of respondents’ address, marital status, number of children if any, education, employment status, and income level. The dataset also contains responses to every individual question on the survey instrument. There is also a field for the open ended question at the end of the survey that asks : “Please describe the biggest obstacles for you in your diet.” These responses are provided in raw form, as verbatim responses.

**Table 1:** List of data files

Label	Name of data file/data set	File types (file extension)	Data repository and identifier (DOI or accession number)	Reference number
Data file 1	Data identification number for dataset:	*MS Excel file (.xlsx)*	Repository name: Synapse 10.7303/syn38269688.1	[1]
Data file 2	Data identification number for survey instrument:	*MS Excel file (.xlsx)*	Repository name: Synapse 10.7303/syn38288698.1	[2]

Direct URL to data: https://www.synapse.org/#!Synapse:syn38269688.1/datasets/

## Limitations

The limitations of these data are these inherent to any dataset based on survey responses. In particular, respondents with biases may select themselves into the sample. Nonetheless, these data could be useful to understand the barriers to healthy eating amongst young adults. These data could be used to design targeted solutions for young adults who struggle to meet recommended dietary guidelines.

## Data Availability

The data described in this Data note can be freely and openly accessed on *[Synapse]* under *[DOI*10.7303/syn38269688.1*]* after registration as a synapse user at http://synpase.org. Please see Table 1 and references [1, 2] for details links to the data.
